# B-cell populations are expanded in breast cancer patients compared with healthy controls

**DOI:** 10.1007/s12282-017-0824-6

**Published:** 2017-12-04

**Authors:** Banri Tsuda, Asuka Miyamoto, Kozue Yokoyama, Rin Ogiya, Risa Oshitanai, Mayako Terao, Toru Morioka, Naoki Niikura, Takuho Okamura, Hirohito Miyako, Yuki Saito, Yasuhiro Suzuki, Yoshie Kametani, Yutaka Tokuda

**Affiliations:** 1 Department of Breast and Endocrine Surgery, Tokai University School of Medicine, 143 Shimokasuya, Isehara, Kanagawa, 259-1193 Japan; 2 Department of Breast and Endocrine Surgery, Tokai University Hachioji Hospital, Hachioji, Japan; 3 Department of Surgery, Niwa Hospital, Odawara, Japan; 4 Department of Molecular Life Science, Division of Basic Medical Science, Tokai University School of Medicine, Isehara, Japan

**Keywords:** B cell, Breast cancer, FACS, HER2, Memory B cell

## Abstract

**Background:**

Historically, humoral immunity was considered unimportant in anti-tumor immunity, and the differentiation and anti-tumor activity of B cells in breast cancer are poorly understood. However, it was recently discovered that B cells participate in tumor immunity through both antibody production and immunosuppressive mechanisms. We analyzed the expression of B-cell differentiation markers in detail using fluorescence-activated cell sorting to investigate the relationship between B-cell subsets and breast cancer etiology.

**Methods:**

Blood samples were taken from breast cancer patients and healthy donors, and peripheral blood mononuclear cells were collected. B cells at various stages of differentiation were identified by the expression of combinations of the cell surface markers CD5, CD19, CD21, CD24, CD27, CD38, CD45, and IgD. Statistical analysis of the proportions of each B-cell subtype in the different patient groups was then performed.

**Results:**

Twenty-seven breast cancer patients and 12 controls were considered. The proportion of total B cells was significantly higher in cancer patients than in controls (11.51 ± 2.059 vs 8.905 ± 0.379%, respectively; *p* = 0.001). Breast cancer patients were then classified as High-B or Low-B for further analysis. A significantly higher proportion of memory B cells was found in the High-B group than in the Low-B or control groups (*p* = 0.003 and *p* = 0.043, respectively).

**Conclusions:**

Breast cancer patients generally have a higher proportion of B cells than healthy controls, but this is highly variable. Analysis of the major B-cell surface markers indicates that memory B cells in particular are significantly expanded, or more robust, in breast cancer patients.

**Electronic supplementary material:**

The online version of this article (10.1007/s12282-017-0824-6) contains supplementary material, which is available to authorized users.

## Introduction

Immunotherapeutic approaches such as peptide vaccines have been used to stimulate the host’s intrinsic immune response to cancer [1]. Previously, we developed the novel anti-HER2 peptide vaccine CH401MAP following an in vitro analysis of T cells from Japanese breast cancer (BC) patients that revealed lower proportions of both killer and helper T cells in BC patients compared with healthy donors [2, 3]. While the importance of the relative quantities of T-cell subsets is very clear in cancer progression, with many groups, including ours, reporting studies concerning their impact [4], there have been no detailed reports of the anti-tumor effects of B cells to date. Indeed, it has been argued that T cells are the most important cellular anti-tumor effectors, and that humoral immunity does not play an important role in tumor rejection [5]. B cells carry specific antibodies on their cell surface that enable them to recognize specific antigens, and may then differentiate into either memory B cells or antibody-producing plasma cells that are capable of producing large quantities of antibodies when the cells encounter their specific antigens for a second time. Additionally, when presenting specific antigens to T cells, B cells secrete immunoregulatory cytokines to modulate cellular immunity and activate antibody production. Furthermore, there is a growing appreciation of the role of B cells in cancer development [6].

Recently, regulatory B cells producing IL-10 have been reported [7], initially in the context of responses to autoimmune and infectious diseases. Evans et al. reported that B cells can be both etiologic agents and suppressors of disease, and showed that splenic T2 marginal zone B-cell precursors were the major IL-10-producing B cells in a murine model of collagen-induced arthritis [8]. IL-10 expression was also detected in splenic plasma cells from Salmonella-infected mice [9]. More recently, B cells have been reported to suppress immune responses to cancer, and regulatory B cells in particular have been suggested to be important for this immunosuppression [10]. Conversely, the importance of B-cell migration in anti-tumor immune responses has also been discussed [7, 11], and a role for tumor-specific antibodies in the prevention of tumor recurrence has been suggested [12]. There is, therefore, a growing body of evidence to suggest that B cells do participate, both positively and negatively, in anti-tumor immune responses.

We hypothesized that the B-cell subsets that develop within the tissues of BC patients and healthy controls will differ, as was seen in experiments examining T-cell subsets. We, therefore, performed fluorescence-activated cell sorting (FACS) analysis of peripheral blood mononuclear cells (PBMCs) from 27 BC patients and 12 healthy donors, and analyzed in detail the expression of B-cell differentiation markers and thus the proportion of each cellular subset.

## Materials and methods

### Selection of patients and healthy donors

When selecting BC patients, 27 women of 20 years of age or older, who displayed histologically confirmed breast cancer but no history of malignant disease and who had not yet undergone surgery, were enrolled at Tokai University Hospital in Kanagawa, Japan, between September 2013 and November 2014. The control group consisted of twelve healthy female donors with no history of malignant disease. The design of this study was approved by the Tokai University Hospital Institutional Review Board, and met all the ethical standards defined in the declaration of Helsinki. All participants gave informed consent prior to the study’s commencement.

### Immunohistochemistry to define study groups

“Hormone receptor and HER2 receptor status was determined using immunohistochemistry analysis. In case of an uncertain result for the HER2 receptor status, fluorescent in situ hybridization (FISH) analysis was used to clarify the status.”

### Sample processing

Blood samples (7.5 ml) were collected both from healthy donors and from patients on the morning of their surgery using Vacutainer ACD tubes (NIPRO Corporation, Japan, Osaka). PBMCs were then isolated from the collected blood by density centrifugation at 5000*g* for 30 min at 20 °C using Ficoll-Hypaque reagent (Sigma-Aldrich, London, UK) according to the manufacturer’s instructions. PBMCs were aspirated and washed with phosphate-buffered saline at 3000*g* for 5 min at 4 °C. All samples were processed within 12 h of collection.

### Immunofluorescence staining and flow cytometry analysis

The antibodies used in B-cell staining and characterization methods are described below. PE/Cy7-conjugated anti-human CD5 antibody (clone UCHT2), APC/Cy7-conjugated anti-human CD19 antibody (clone HIB19), PerCP/Cy5.5-conjugated anti-human CD24 antibody (clone ML5), Alexa Fluor 700-conjugated anti-human CD38 antibody (clone HIT2), and Pacific Blue-conjugated anti-human CD45 antibody (clone HI30) were purchased from BioLegend (San Diego, CA, USA). PE-conjugated anti-human CD27 antibody (clone M-T271) and FITC-conjugated anti-human IgD antibody (clone IA6-2 (were purchased from BD Bioscience (Franklin Lakes, NJ, USA). APC-conjugated anti-human CD21 antibody (clone FAB4909A) was purchased from R&D Systems (Minneapolis, MN, USA).

Immunofluorescent staining was performed according to previously reported protocols [2, 3], using Fixation/Permeabilization Concentrate, Fixation/Permeabilization Diluent, and Permeabilization buffer (10×) from BD Biosciences (CA, USA).

Cell surface protein expression was examined using flow cytometry. The fluorescence intensity of fluorochrome-labeled cells was measured using a BD Fortessa flow cytometer (BD Biosciences) and FlowJo software version 7.6.1 (Tree Star, Inc. Ashland, Oregon). Data were first gated on the lymphocyte population before subsequent analyses were performed.

### Statistical analyses

The statistical significance of differences between patient groups was assessed with an unpaired two-tailed Student’s *t* test, performed using Microsoft Excel version 14.7.1. Values of *p* < 0.05 were considered statistically significant. All data are presented as the mean ± standard deviation of the mean.

## Results

### Histopathological characteristics of patients with BC patients

For this study, 27 BC patients and 12 healthy donors were enrolled, with mean ages of 59.2 ± 10.8 and 37.6 ± 13.9 years, respectively (Table 1). Histological analysis of the BC patients revealed one ductal carcinoma in situ (DCIS), one invasive lobular carcinoma, and 25 invasive ductal carcinoma cases. Table 1 shows the health status of the HDs and the patient’s cancer status (age, ER, PgR, HER2, Ki-67, Histology and LN metastasis status). The size of the tumor and the status of individual patients, HDs are described in supplemental table 1.

**Table 1:**
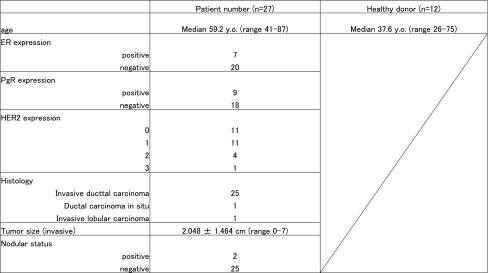
Baseline characteristics of breast cancer patient and healthy donor

### Analysis of B-cell populations using immunofluorescence staining and FACS

**Fig. 1:**
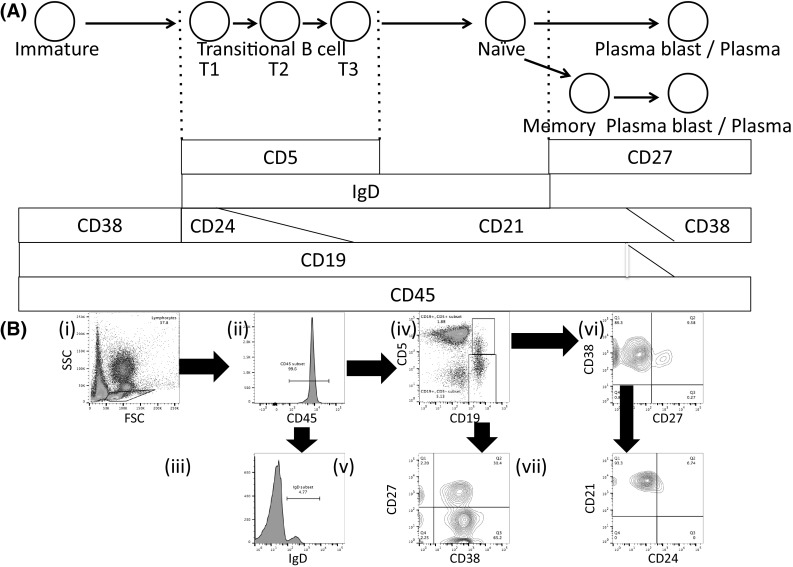
Identifying B-cell subsets using surface marker profiles and fluorescence-activated cell sorting (FACS). **a** Schematic showing cellular subsets during B-cell differentiation and the cell surface markers that are expressed on the corresponding B-cell subsets. Firstly, immature B cells enter the bloodstream from the bone marrow before eventually reaching the central arterioles and then the marginal zone (MZ) of the spleen. The immature B cells then become transitional B cells, and are classified as either T1, T2, or T3. T1 B cells express CXCR5 on their surface and migrate from the MZ region to the B region of the spleen by interacting with CXCL13 secreted by follicular dendritic cells, and mature into T2 and then T3 B cells before leaving the spleen as mature, naive B cells. Naive B cells have yet to recognize antigen and comprise approximately 60% of peripheral blood B cells. These cells are characterized as CD27^−^ and recognize antigens through IgM- and IgD-type receptors. When naive B cells migrate to the lymph nodes, where they recognize antigens, they then differentiate at the germinal center to become memory B cells, expressing cell surface IgG, IgA, and IgE, or, occasionally, IgM. Naive B cells may also be activated to differentiate directly into antibody-producing plasma cells. Finally, when memory B cells circulating in the peripheral blood encounter an antigen for a second time, they may become a plasma cell and rapidly produce large numbers of high affinity antibodies. The common white blood cell antigen CD45 and the B-cell marker CD19 are expressed at all stages from immature B cell to plasmablast. Transitional B cells are reported to express CD5 [26]. While CD24 is expressed at the more immature T1/T2 stages, CD21 expression increases as the cells mature towards the T2/T3 stage. Mature naive B cells express high levels of IgD, memory B cells express CD27, and antibody-producing plasma cells express CD27 and CD38. **b** Representative FACS data depicting normal PBMCs taken from a 49-year-old woman. The arrow denotes the fraction that has been expanded. (i) Firstly, forward scatter (FSC) and side scatter (SSC) were measured, and the lymphocyte gate was defined. (ii) CD45^+^ cells were selected from the lymphocyte fraction. (iii) Cells highly positive for both IgD and CD45 were defined as Naive B cells. (iv) CD45^+^ cells were selected and then CD19 (horizontal axis) and CD5 (vertical axis) positivity was assessed. (v) CD45^+^/CD19^+^/CD5^−^ cells were selected and then the CD38 and CD27 positivity of this subset, shown on the horizontal and vertical axes, respectively, was assessed. CD45^+^/CD19^+^/CD5^−^/CD38^+^ cells were defined as plasmablast cells and CD45^+^/CD19^+^/CD5^−^/CD27^+^ cells were defined as memory B cells. (vi) CD45^+^/CD19^+^/CD5^+^ cells were selected and then the CD38 and CD27 positivity of this subset, shown on the vertical and horizontal axes, respectively, was assessed. (vii) CD45^+^/CD19^+^/CD5^+^/CD38^+^/CD27^−^ cells were selected and levels of CD24 and CD21 positivity, shown on the horizontal and vertical axes, respectively, was assessed. The CD24^+^ and CD21^+^ cells were defined as T1 and T3 transitional B cells, respectively

The relative proportions of B-cell subsets were analyzed in detail in BC patients and healthy donors. The cell populations found throughout the B-cell differentiation process, and the cell surface markers that define each differentiation stage, are illustrated in Fig. 1a. Flow cytometry data identifying each B-cell population, defined using these markers, was collected for all study participants, and representative data from a healthy 49-year-old patient is shown in Fig. 1b. The B-cell data were obtained firstly by gating on lymphocytes and then selecting the CD45^+^/CD19^+^ cells. Sequential gating was then used to identify and quantify B-cell subsets at different stages of differentiation. The specific combinations of surface markers used to identify each subset are described in the Fig. 1b legend.

**Fig. 2:**
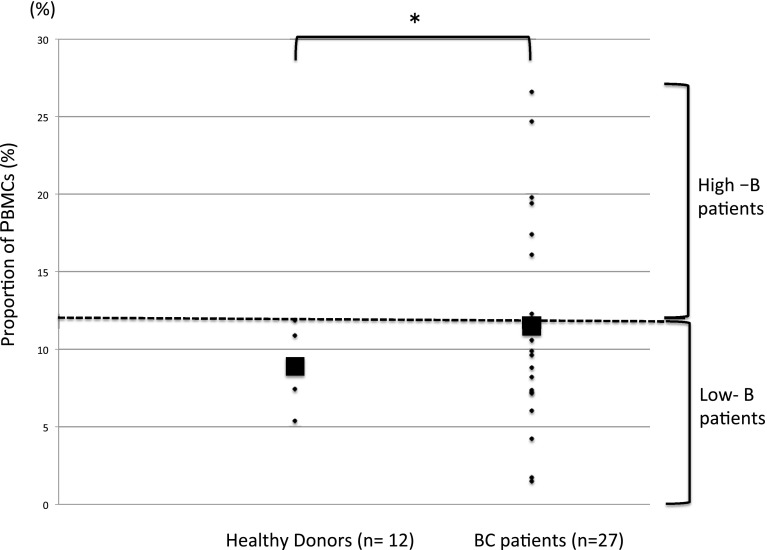
Strip chart showing the proportion of total PMBCs that are defined as B cells (CD45^+^/CD19^+^) in healthy donors and BC patients. The filled square denotes the group medians. The dashed line marks the highest recorded proportion of B cells in the healthy controls, and defines the boundary between the ‘High-B’ and ‘Low-B’ groups. *p < 0.05

Using the cell surface markers indicated in Fig. 1, profiles of the relative proportions of B-cell subsets at each differentiation stage were analyzed for BC patients versus healthy controls (Table 2). Overall, a significantly higher proportion of PBMCs were B cells (CD45^+^/CD19^+^) in BC patients than in healthy donors (11.51 ± 2.059 and 8.905 ± 0.379%, respectively; *p* = 0.001), with more variation seen in the patient group (Table 2 and Fig. 2). Conversely, while the proportion of B cells at each differentiation stage was generally higher in BC patients, these differences were not statistically significant (Table 2).

**Table 2:** Profiles of the relative proportions of B-cell subsets at each differentiation stage

	Healthy donors (%) (*n* = 12)	Breast cancer patient (%) (*n* = 27)	*p* value
B cell	8.905 ± 0.379	11.51 ± 2.059	0.001*
T1	0.113 ± 0.080	0.240 ± 0.346	0.065
T3	1.926 ± 1.585	2.340 ± 4.013	0.287
Naïve	5.790 ± 2.759	7.095 ± 5.481	0.383
Memory	3.265 ± 1.932	4.840 ± 3.560	0.069
Plasma	2.407 ± 1.061	2.215 ± 1.300	0.618
Others	6.170 ± 0.378	1.186 ± 2.059	0.141

Values represent mean ± SD. *p* values were calculated with an unpaired two-tailed Student’s *t* test. *p < 0.05

To further investigate B-cell characteristics in the BC group, patients were designated either ‘High-B’ or ‘Low-B’ according to the proportion of B cells within the PBMC population, with ‘High-B’ being defined as those above the highest recorded B-cell proportion in healthy donors (Fig. 2). The proportion of each cell subset for the two patient groups and statistical analyses of the differences between the groups are shown in Table 3.

**Table 3:** Comparison of the ratio of B cells between HD, High-B and Low-B at each stage

	Healthy donors (%) (*n* = 12)	High-B (%) (*n* = 12)	Low-B (%) (*n* = 15)	*p* value		
				HD/high-B	HD/low-B	High-B/low-B
B cell	8.905 ± 0.379	12.12 ± 2.782	9.204 ± 0.140	0.024*	0.04*	< 0.001*
T1	0.113 ± 0.080	0.246 ± 0.216	0.160 ± 0.202	0.111	0.493	0.333
T3	1.926 ± 1.585	4.488 ± 5.642	1.458 ± 1.185	0.193	0.499	0.125
Naïve	5.790 ± 2.759	7.638 ± 4.181	5.180 ± 3.270	0.151	0.867	0.137
Memory	3.265 ± 1.932	5.649 ± 2.409	3.566 ± 2.567	0.003*	0.309	0.043*
Plasma	2.407 ± 1.061	2.463 ± 1.240	2.126 ± 1.360	0.913	0.559	0.53
Others	6.170 ± 0.378	2.738 ± 2.495	1.611 ± 1.772	0.247	0.432	0.238

Values represent mean ± SD. *p* values were calculated with an unpaired two-tailed Student’s *t* test. *p < 0.05

As expected, there were significantly more B cells in the PBMC population of the High-B group than in the controls or the Low-B group, and statistically fewer B cells in the Low-B group than in the controls. No significant differences were seen in the proportions of T1 or T3 transitional B cells, naive B cells, or plasma cells between any of the groups. Interestingly, however, while the proportion of memory B cells present in the healthy donor and Low-B groups were equivalent (3.265 ± 1.932 and 3.566 ± 2.567%, respectively; *p* = 0.309), significantly more memory B cells were seen in the High-B group than in the other groups (5.649 ± 2.409%; *p* = 0.003 vs healthy donors, and *p* = 0.043 vs Low-B group).

**Fig. 3:**
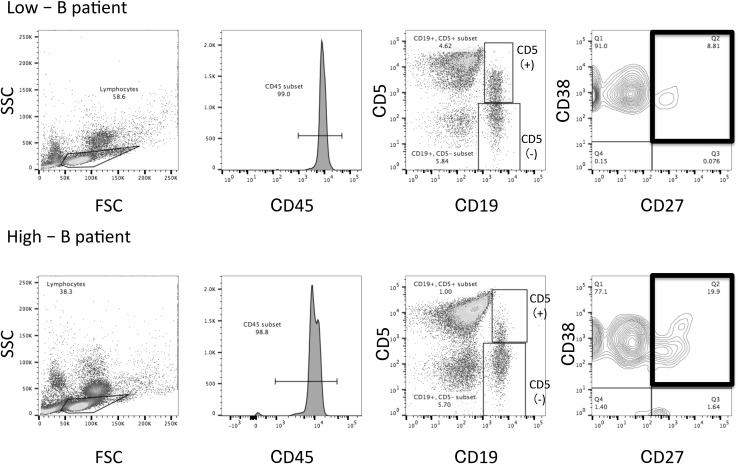
Representative FACS data showing the identification of memory B cells in a High-B group patient (**a**) or a Low-B group patient (**b**). The cell surface markers being detected are noted in the axis labels. Black square: gate on memory B cells, defined as CD45^+^/CD19^+^/CD5^−^/CD38^+^/CD27^+^. Inset numbers refer to the proportion of total PBMCs within each gate. *SSC* side scatter. *FSC* forward scatter

Representative FACS data for the High-B and Low-B groups are shown in Fig. 3. The Low-B patient was a 65-year-old HER2 0 group woman with a 16 × 15 mm lesion of the scirrhous subtype of invasive ductal carcinoma, no lymph node metastasis and immunohistochemistry staining showing 90% ER, 80% PgR, and 5% Ki-67. The High-B patient was a 67-year-old HER2 1 + group woman, with a 15 × 15 mm lesion of the papillotubular subtype of invasive ductal carcinoma, no lymph node metastasis, and immunohistochemistry staining also showing 90% ER, 80% PgR, and 5% Ki-67. The major differences in memory B-cell numbers between the two patients are highlighted by black boxes (Fig. 3). Although the clinical data were similar, the proportion of memory B cells in the respective total PBMC populations varied greatly.

## Discussion

In this study, we found differences in B-cell differentiation between BC patients and healthy donors. The proportion of total B cells was significantly higher in BC patients than in controls, although no differences in the relative proportion of each subset were observed. The proportion of B cells in BC patients ranged widely, and we classified BC patients into High-B or Low-B groups depending on their B-cell proportion, with High-B being defined as values above the highest B-cell proportion observed in healthy controls. Interestingly, the proportion of memory B cells in the High-B group was significantly higher than in either the Low-B BC group or the healthy donor controls. In this study, the upper limits of HDs without cancer history were classified as Low-B and High-B groups using threshold values. Whether this classification is the best is currently not known.

Cancer immunity is mainly conferred by the natural killer cells and natural killer T cells of the innate immune system, by helper and cytotoxic T cells, and by humoral immunity via antibody-producing B cells [13]. The relative contributions of innate and acquired immunity have been well studied and continue to be debated [10, 14–16].

The effectiveness of antibody-based therapies, including trastuzumab and bevacizumab, in breast cancer is well established, and the importance of the immune system in cancer development was highlighted once again by studies using inhibitors of immune checkpoint pathway components such as PD-1, PD-L1, and CTLA-4. Cancer cells can co-opt these checkpoints to evade the immune system, and immune checkpoint inhibitors act to override these immune blockades and thus enhance cancer immunity [17].

Immune checkpoint inhibitors in combination with peptide vaccines are predicted to accelerate the anti-tumor activity of the immune system, although the peptide vaccines GP2 and AE75, designed to stimulate cytotoxic T cells, have thus far only succeeded in preventing the recurrence of cancer [18, 19]. Indeed, peptide vaccines that activate only T cells may trigger the expression of cytokines such as IL-2 and IFN-γ in T cells, but will not overcome the immune checkpoint blocks induced by PD-1 and PD-L1 expression. However, increased tumor-specific antibody production through the activation of B cells in combination with checkpoint inhibitors provides an as yet poorly studied potential new treatment strategy.

Previously, we reported preclinical research into a novel anti-HER2 peptide vaccine named CH401MAP that could both increase T-cell proliferation and the proportion of activated T cells, and trigger specific antibody production from B cells [2, 3]. Unlike previous peptide vaccines, a unique and promising feature of CH401MAP is its ability to stimulate both T cells and B cells simultaneously, as well as its potential utility in BC patients with a wide range of HLA types.

In the present study, we report that the total B-cell population was significantly increased in the BC patient group compared to controls, indicating that B cells may have a role to play in tumor promotion. The number of WBCs of the patients ranged from 4000 to 9000, which is not different from the normal range in HDs. Therefore, we focused on the ratio of B-cell subsets in PBMC population, because the condition of the immunity of patients was reflected in the cellularity. Therefore, we found correlation between age and B-cell ratio in HD group, BC patient group, HD + BC patient group (supplemental figure 1). However, there was no correlation between age and B-cell ratio in all groups. Considering the two patient groups identified, Low-B patients had the same proportion of B cells as the control group, while the proportion of B cells in High-B patients was higher than in the controls. Comparative statistical analysis revealed this B-cell increase to be due to the significantly increased proliferation or survival of memory B cells in the High-B group compared with the other groups. The increase in B cell in BC patients, and the increase in memory B cells in particular, is indicative of previous humoral immunity activation in BC patients, although the cause of this and its implications for cancer progression cannot be determined in this study. If the expanded memory B cells are tumor antigen-specific, then activation of these cells would be expected to suppress cancer development; conversely, if the expanded B-cell populations act to suppress T-cell responses through the production of cytokines such as IL-10, this could actually drive cancer progression.

Previously, it was impossible to analyze B-cell differentiation without using in vivo systems [20]. It is now possible to perform detailed analyses using only flow cytometry, although more comprehensive analysis of the cellular differentiation process will likely require the use of complementary methods. However, the manpower and cost involved in collecting multiple samples should be considered, and the approach used here, using specific subsets of cell surface markers, is an effective and practical alternative to advanced staining techniques.

In the future, it will be interesting to analyze B-cell activation and differentiation in BC patients following the in vitro stimulation of PBMCs with peptide vaccines, as a corresponding increase in the proportion of B cells at each differentiation stage would be expected. In our study, we found that the proportion of memory B cells is elevated within BC patients, but this is not considered to be an increase in the role of memory. Because they are actually affected by BC. The reason that the ratio of memory B cells increased was the possibility that Breg was included in this fraction. Breg is a cell population defined by CD19 +/CD24^high^/CD38^high^ and IL-10 is defined as Breg [7]. It is possible that Breg was included in the fraction of memory B cells in this analysis. There is a possibility that more interesting results may be obtained by further analyzing in the future. IL-10 secreted by plasmablasts suppresses cancer immunity. Detailed analysis of the B-cell differentiation process, and the subsequent selection of patients who are expected to produce antibodies following peptide vaccine administration, could lead to enhanced antitumor immunity in some cases.

The antitumor effects of peptide vaccine administration and side effects of the peptide vaccine, as well as the progression of the tumor, must be considered when deciding treatment, and the risks and benefits of treatment must be carefully considered. Appropriate treatment decisions depend on many factors, including clinical diagnosis, staging and immune status. In this study, the number of patients was limited, and the stage and subtype of BC were not considered. Additionally, the mechanisms underlying the differences in B-cell subsets between the groups are unclear. In future, it will be necessary to conduct more detailed analyses on a larger number of clinical samples. Presently, several TILs have been detected in TNBC and HER 2 types [21–25]. Further analysis of TIL, in this study, may form a basis for understanding the tumor microenvironment in detail.

In summary, we show that the proportion of B cells among PBMCs was significantly higher in BC patients compared to healthy donors, but that this proportion of B cells within the BC patient group was highly variable. Furthermore, memory B cells were significantly enriched in the group of patients with the highest proportion of B cells, as determined using the simple and practical method developed in this study that involves the staining of major cell surface markers. Multicolor FACS analysis could, therefore, represent a powerful tool in the detection of patients B-cell status.

## Electronic supplementary material

Below is the link to the electronic supplementary material.

Supplemental Figure 1 The correlation between age and B-cell ratio, (A) BC patients group, R2 = 0.00237, (B) HD group, R2 = 0.07173, (C) BC patients and HD group, R2 = 0.03542. (PDF 52 kb)

Supplementary material 2 (PDF 44 kb)
