# Supplementary figures and images for: B-cell populations are expanded in breast cancer patients compared with healthy controls
Source: Breast Cancer. 2017 Dec 4;25(3):284–91. doi: 10.1007/s12282-017-0824-6 (PMC5906508; doi:10.1007/s12282-017-0824-6)

(A) BC patients

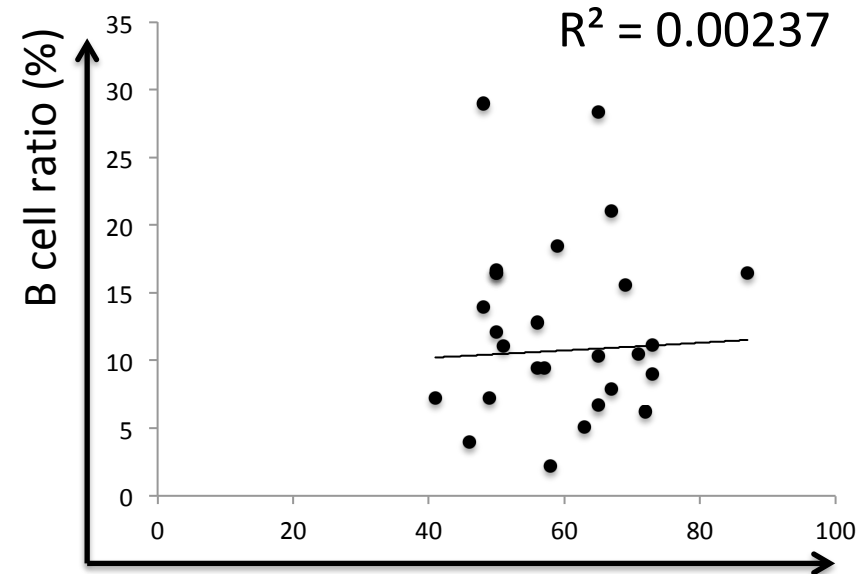

(B) HD

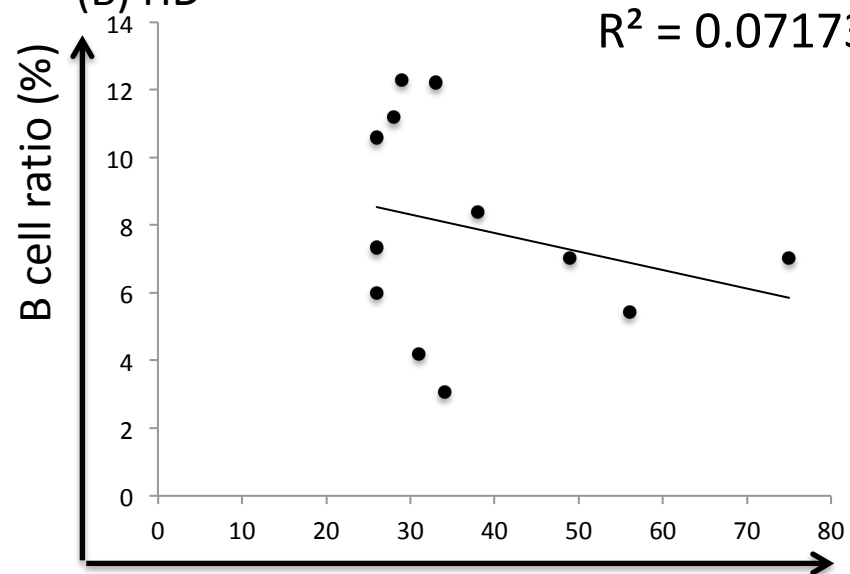

(C) BC patients and HD

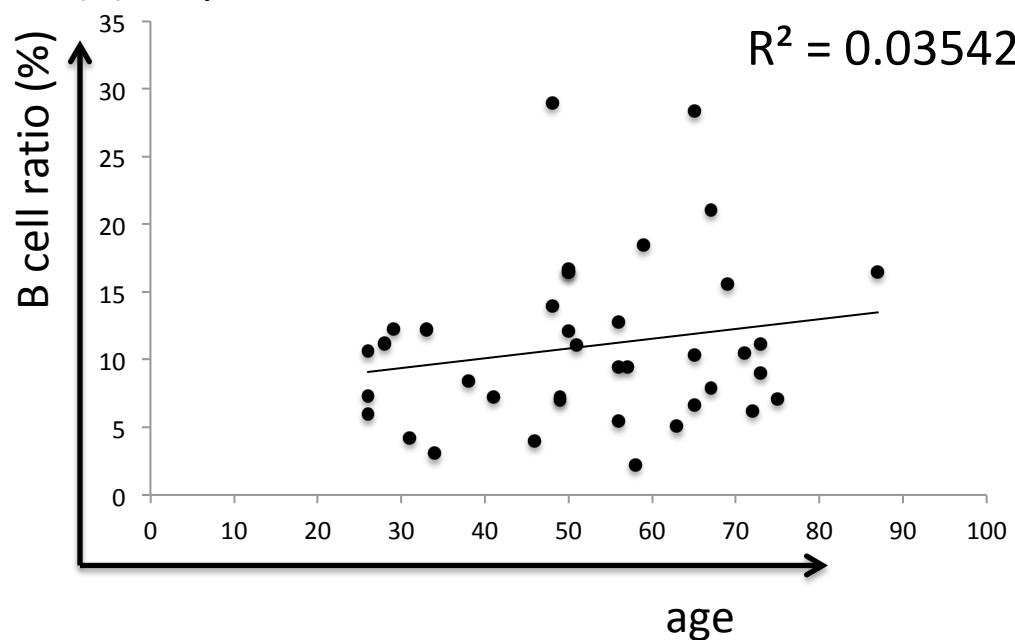

Supplement: Supplementary file 1 — Supplemental Figure 1 The correlation between age and B-cell ratio, (A) BC patients group, R2 = 0.00237, (B) HD group, R2 = 0.07173, (C) BC patients and HD group, R2 = 0.03542. (PDF 52 kb) [file 12282_2017_824_MOESM1_ESM.pdf]
