# Supplementary material for: B-cell populations are expanded in breast cancer patients compared with healthy controls
Source: Breast Cancer. 2017 Dec 4;25(3):284–91. doi: 10.1007/s12282-017-0824-6 (PMC5906508; doi:10.1007/s12282-017-0824-6)
Supplement: Supplementary file 2 — Supplementary material 2 (PDF 44 kb) [file 12282_2017_824_MOESM2_ESM.pdf]

patient No.	Age (y/o)	Pathological classification	Tumor size (mm)	LN metastasis	ER expression (%)	PgR expression(%)	HER2 score(IHC)	Ki67 (%)	B cell ratio (%)	T1 ratio (%)	T3 ratio (%)	Naïve B cell ratio (%)	Memory B cell ratio (%)	Plasma cell ratio (%)
1	65	scirrhous	30x25	−	90	0	1	20	28.35	2.71	97.3	3.19	71.1	22.8
2	59	mucinous	25x15	−	90	30	0	10	18.5	9.86	90.1	5.7	75.3	22.4
3	50	papillotubular	12x12	−	90	90	1	5	12.08	9.55	89.9	3.26	73.6	22.8
4	50	mucinous	20x10	−	90	40	1	10	16.49	1.82	98.1	12.1	67.2	26.2
5	56	papillotubular	10x10	−	0	0	1	10	12.78	1.69	98.3	8.07	63.5	32.6
6	57	scirrhous	28x14	−	0	0	0	50	9.43	3.95	94.4	6.6	73.8	22.9
7	71	papillotubular	20x15	+	0	0	0	20	10.46	8.01	92	5.73	43.5	52.2
8	67	papillotubular	15x15	−	90	80	1	5	21.08	2.6	97.3	16.8	65.1	30.2
9	58	scirrhous	30x25	−	100	5	0	10	2.23	32.7	67.2	1.52	62.8	33.4
10	73	mucinous	65x70x	+	90	60	1	10	11.14	11.1	88.9	6.68	44.6	49.5
11	72	mucinous	17x10	−	0	0	0	20	6.23	0.79	91.3	3.72	70.2	26.5
12	67	mucinous	22x10	−	0	0	1	5	7.88	3.74	95.6	5.39	60.3	36.2
13	51	papillotubular	10x10	−	0	0	0	5	11.04	16.3	83.4	7.06	35.3	62.6
14	48	papillotubular	16x15	−	70	60	1	5	13.96	0.91	99	10.8	75.2	20.7
15	65	papillotubular	(61x59)us	−	90	70	0	10	10.35	16.7	82.6	6.51	50	48
16	48	papillotubular	15x5	−	10	5	0	5	29	15	84.7	26	93.4	5.44
17	73	mucinous	15x10	−	90	80	0	20	8.99	6.19	93.8	5.92	39	57.8
18	46	DCIS	0	−	70	70	3	5	3.94	1.45	98.1	3.09	76.7	21.4
19	41	papillotubular	7x7	−	90	90	0	5	7.26	12.3	86.8	5.2	65.2	28.6
20	49	papillotubular	16x12	−	70	70	2	5	7.26	16.2	83.5	4.48	51.5	40.9
21	56	papillotubular	11x10	−	90	10	2	1	9.45	5.97	93.6	6.35	77	15.3
22	50	mucinous	20x10	−	90	40	1	10	16.49	1.82	98.1	12.1	67.2	26.2
23	65	scirrhous	16x15	−	90	80	0	5	6.66	6.78	92.4	5.74	86.4	10.9
24	50	mucinous	20x10	−	90	40	1	10	16.65	9.73	90.2	12.9	71.3	21.5
25	69	apocrine	16x16	−	0	0	2	20	15.6	9.91	89.9	10.6	53.9	44.2
26	63	ILC	14x5	0	90	90	2	5	5.06	3.34	94.3	16.5	76.9	20.1
27	87	papillotubular	(17x16)us	−	90	0	1	20	16.49	1.82	98.1	12.1	67.2	26.2
HD														
1	26								10.6	26.1	73.6	5.72	45.1	52.6
2	26								7.35	6.78	93.2	3.77	46.2	48
3	33								12.22	1	99	8.89	57.1	37.2
4	75								7.04	6.7	93.3	4.8	65	30.6
5	56								5.42	17.1	82.9	3.46	69.6	28
6	26								5.99	14	85.1	3.78	77.1	20.1
7	31								4.2	14.6	85.4	3.22	59.2	38.6
8	34								3.06	11.7	88.3	2.41	46.5	47.6
9	49								7.03	8.7	91.3	4.16	41	53.7
10	28								11.18	0.88	99	8.17	46.9	46
11	38								8.4	2.04	97.8	6.04	54.5	41.7
12	29								12.3	3.61	96.4	9.56	36.1	59.3

Supplemental table 1: The age, tissue type, tumor diameter, lymph node metastasis, ER, PgR, HER2, Ki-67, ratio of differentiation stages of B cells in each patient.
